# Air All in the Wrong Places: A Case of Pneumorrhachis Secondary to Chronic Vomiting

**DOI:** 10.7759/cureus.37501

**Published:** 2023-04-12

**Authors:** Nicholas R Rotsching, Jay Mathias, Marc Gutierrez, Nathaniel Ford, James Lamb

**Affiliations:** 1 Internal Medicine, Wright State University, Dayton, USA; 2 Internal Medicine, Wright Patterson Air Force Base, Dayton, USA

**Keywords:** intractable vomiting, diabetes, pneumomediastinum, gastroparesis, pneumorrhachis

## Abstract

Pneumorrhachis (PR) is a rare phenomenon in which air is present in the spinal canal. PR can be stratified into different categories based on etiology, with spontaneous PR being the least common. In this report, we describe the case of a 33-year-old male with a four-year history of emesis secondary to chronic gastroparesis who presented with pleuritic chest pain radiating to the neck. A CT scan of the chest showed pneumomediastinum, with air extending into the soft tissues of the neck and the spinal canal. A literature review found a trend between maneuvers that increase intrathoracic pressure, such as emesis or coughing, and the incidence of spontaneous pneumomediastinum, in which air may freely communicate with the epidural space of the spinal canal. Currently, there are no guidelines for the management of patients with PR. From our experience, conservative management of asymptomatic PR is an appropriate approach for these patients.

## Introduction

Pneumorrhachis (PR) is a rare phenomenon in which air is present in the spinal canal. In the literature, reported etiologies fall into one of three categories: traumatic, iatrogenic, or spontaneous [1]. Spontaneous PR is the least common and is believed to occur secondary to maneuvers that increase intrathoracic pressure, such as emesis and coughing [2]. Though spontaneous PR is often asymptomatic, neurologic symptoms have been reported [2-6]. Currently, there are no guidelines for the management of patients with PR. Our case report describes our experience with managing a patient with spontaneous PR and can be used as a resource for managing patients with similar presentations until guidelines are established.

## Case presentation

The patient was a 33-year-old male who presented to the emergency department (ED) with a two-day history of progressively worsening pleuritic chest pain that radiated to the neck with associated dyspnea. He had a past medical history of type 2 diabetes mellitus, hypertension, chronic marijuana use, and chronic nausea and vomiting of a four-year duration that was suspected to be secondary to gastroparesis. A gastric-emptying study had yet to be completed before his current presentation. 

**Figure 1:**
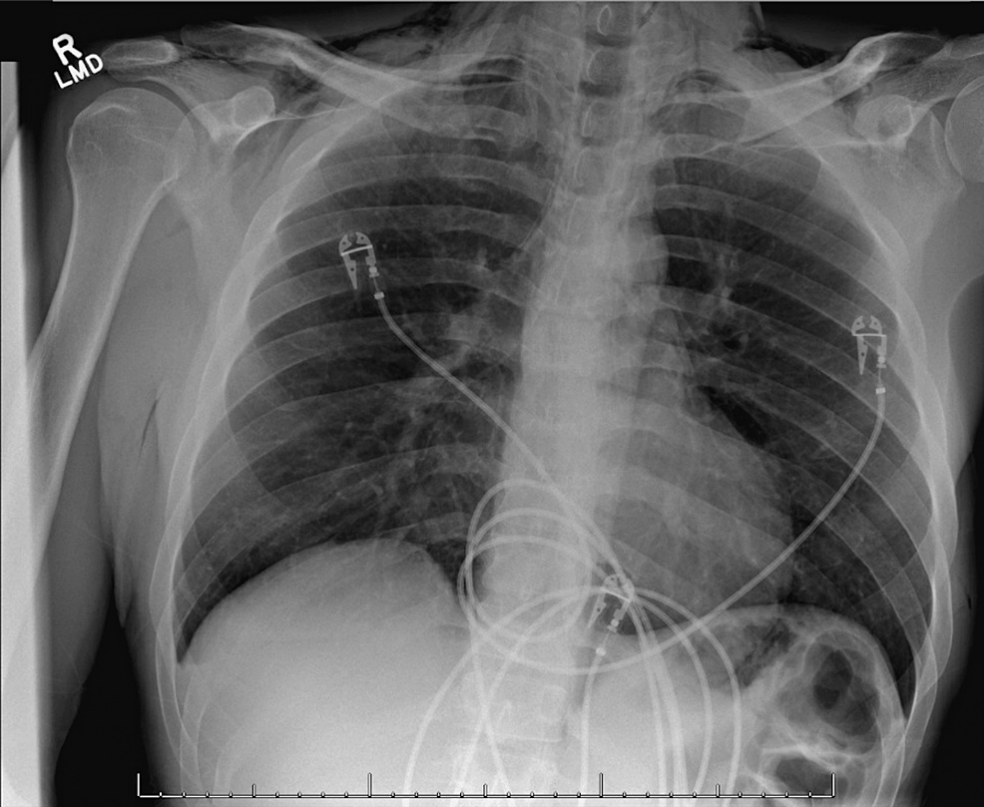
Anterior-posterior chest X-ray demonstrating pneumomediastinum and subcutaneous emphysema

**Figure 2:**
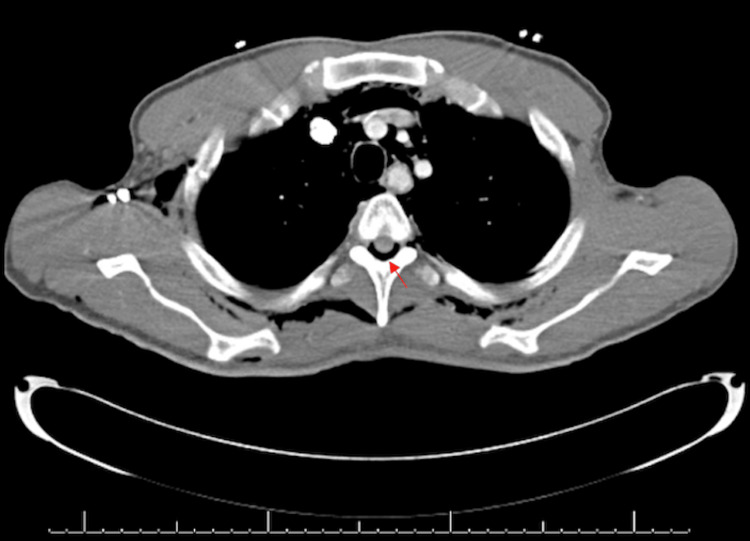
Axial computerized tomography demonstrating pneumorrhachis (red arrow)

**Figure 3:**
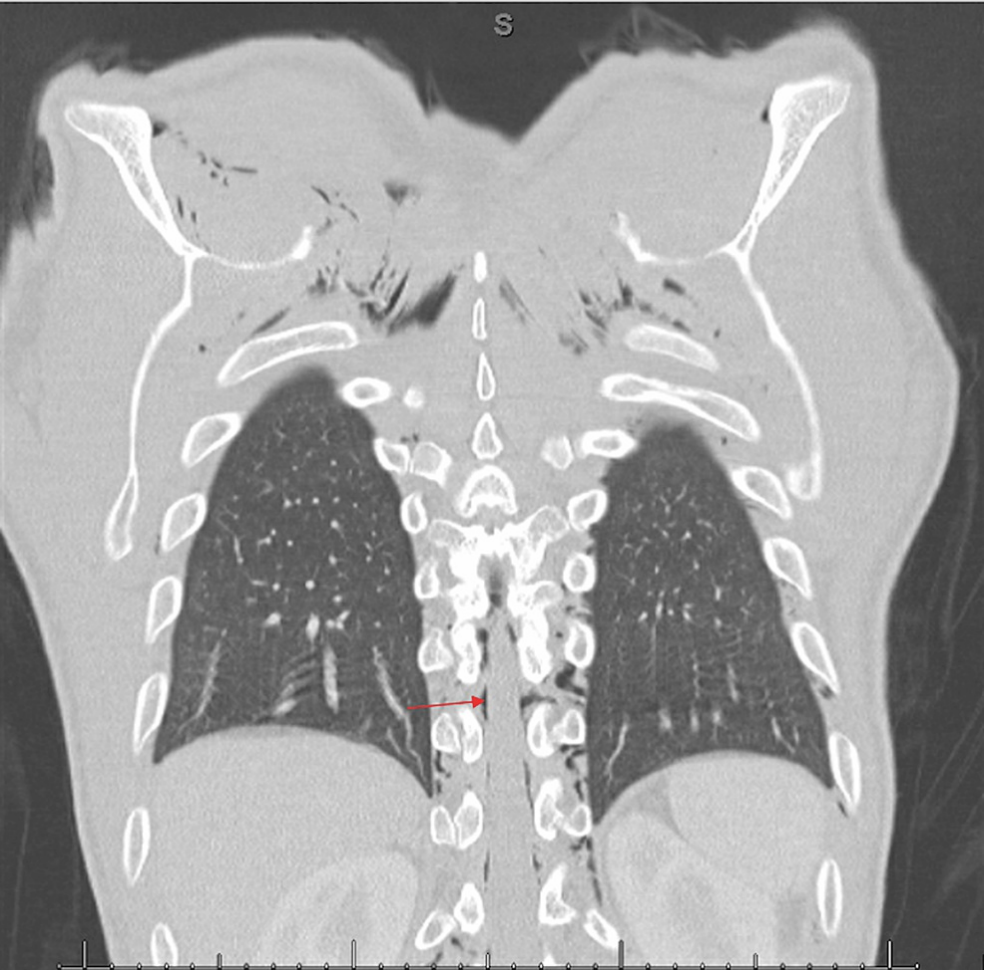
Coronal computerized tomography demonstrating pneumorrhachis (red arrow)

The patient’s physical exam in the ED was remarkable for reproducible tenderness to palpation on the left side of his neck and subcutaneous emphysema in his neck and anterior chest. An electrocardiogram (EKG) showed normal sinus rate and rhythm without ST abnormalities. Furthermore, his troponin, D-dimer, complete blood count, and comprehensive metabolic panel were unremarkable. A chest X-ray (CXR) was obtained, which demonstrated pneumomediastinum, soft tissue emphysema, and a possible trace of left apical pneumothorax (Figure 1). A computerized tomography (CT) of the chest with oral contrast was then performed, which demonstrated no enteric contrast extravasation and ruled out Boerhaave’s syndrome. However, the CT showed extensive pneumomediastinum, with air extending into the soft tissues of the neck, chest wall, and spinal canal (Figures 2, 3). The patient was admitted for further management and evaluation.

**Figure 4:**
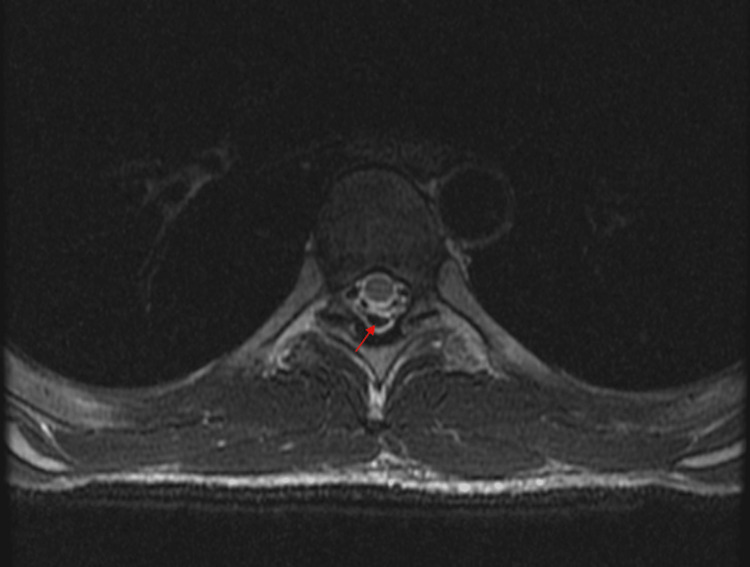
Axial magnetic resonance imaging of the spine demonstrating pneumorrhachis (red arrow)

**Figure 5:**
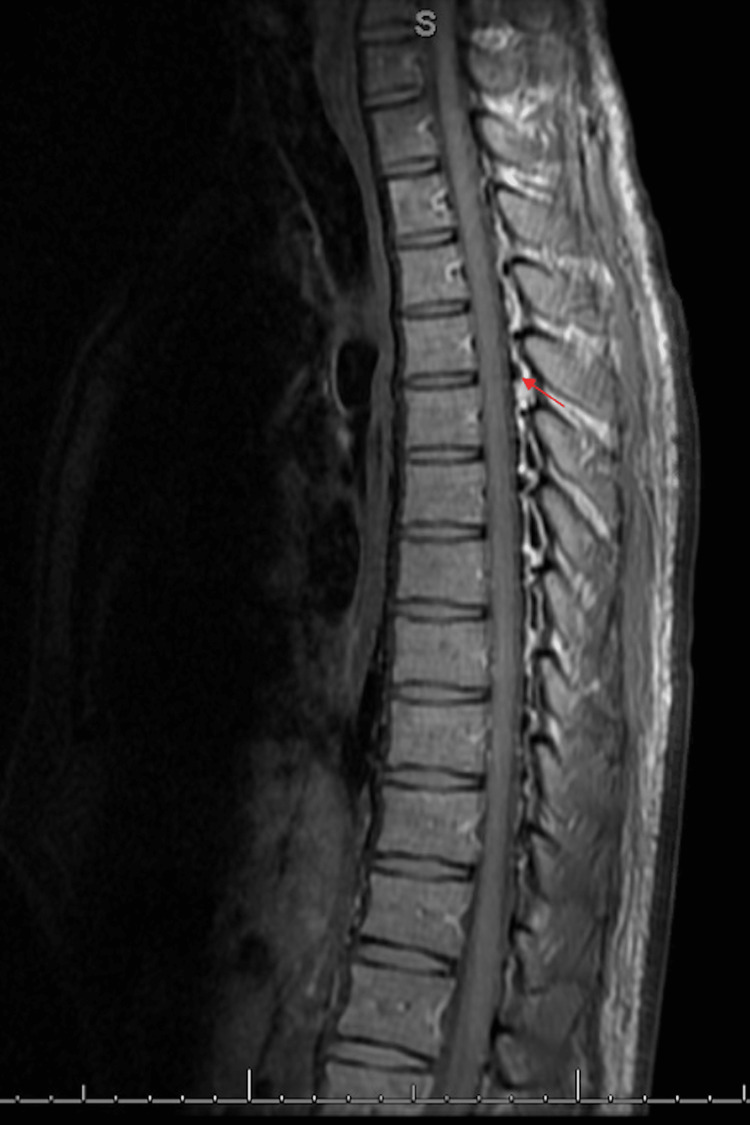
Sagittal magnetic resonance imaging of the spine demonstrating pneumorrhachis (red arrow)

The patient was monitored with serial neurologic exams every four hours and daily CXRs. Additional studies obtained during his hospital stay included a CT head without contrast and magnetic resonance imaging (MRI) of the spine to further evaluate for intracranial air and cord compression, respectively. The CT head demonstrated no acute processes, and the spinal MRI (Figures 4, 5) showed pneumorrhachis of the dorsal thoracic epidural space without significant compromise to the spinal canal. No surgical intervention was indicated, and the patient was managed conservatively with supplemental oxygen, bronchodilators, and medications to avoid maneuvers that would increase intrathoracic pressure, including anti-emetic and anti-tussive medications. The patient was discharged home with his antiemetic regimen, and a close follow-up with his primary care doctor was recommended. An outpatient follow-up CXR four days after discharge showed decreased air in the neck and stable pneumomediastinum compared to the previous exam, a minimal improvement. Two months later, a nuclear medicine gastric emptying study revealed the time to empty half the activity of the stomach to be 408 minutes (the normal range: 50-95 minutes).

## Discussion

Pneumorrhachis (PR) is a rare condition characterized by the presence of air in the spinal canal. In the early 20th century, air was used as a contrast medium in neuroimaging, but its use declined as newer, less toxic contrast agents were utilized. In 1977, Gordon and Hardman reported the first case of non-iatrogenic free air in the spinal canal in a patient who had a motor vehicle accident (MVA). They could only refer to “the traumatic pneumomyelogram” [7]. Newbold et al. [8] first used the term “pneumorrhachis” in 1987, a designation that has since been widely accepted versus other terms such as intraspinal pneumocele, spinal epidural and subarachnoid pneumatosis, and spinal epidural emphysema [8].

The etiology of PR can be divided into three main causes: traumatic, iatrogenic, and spontaneous [1]. Most cases are secondary to traumatic and iatrogenic etiologies [9]. Traumatic injuries to the head, neck, or spine can introduce air into the spinal canal [10-11]. Iatrogenic causes are associated with surgical or anesthetic procedures. Reported triggers of spontaneous PR include forceful coughing, emesis, inhalation of drugs, reactive airway disease, or intense physical exertion. The incidence of spontaneous PR is currently unknown and believed to be underdiagnosed. A 2021 systemic review that identified 49 cases of spontaneous PR published from 1994 to 2021 supports the rarity of this finding [12]. As a result of its pathophysiology (described below), spontaneous PR is commonly found in combination with air in other cavities, such as subcutaneous emphysema, pneumothorax, and/or pneumomediastinum, as seen in our patient [1]. It is reportedly associated with approximately 10% of cases of spontaneous pneumomediastinum, which has an incidence of roughly one in 30,000 patients [13,14].

Macklin et al. [15] described the eponymous Macklin effect, the relationship between maneuvers that increase intrathoracic pressure and spontaneous pneumomediastinum. [15] The elevated intrathoracic pressure caused by these maneuvers increases the bronchoalveolar pressure, resulting in alveolar rupture. Air enters the pulmonary interstitium and travels by the path of least resistance along the peribronchial vascular sheaths toward the hilum and into the mediastinum since the pressure is lower in the mediastinum relative to the lung periphery. [15] From the mediastinum, air can enter the submandibular space, retropharyngeal space, the cervical vascular sheaths due to communicating fascial planes. In 1993, Balachandran et al. [16] proposed that the absence of fascial barriers between the posterior mediastinum and epidural space allowed air to freely travel between these two spaces, resulting in PR [16]. Furthermore, in scenarios of bronchospasm due to the inhalation of drugs or possibly in reactive airway disease, Muller’s maneuver (voluntary inspiration with a closed mouth and obstructed nares) can induce barotrauma from the extreme negative intrathoracic pressure and predispose an individual to develop the above sequelae [17]. Our patient routinely vaped marijuana, a practice that has been shown to induce lung injury with subsequent PR [18]. However, our patient did not have the history or imaging findings to support vaping as the underlying etiology, and we believe his presentation was secondary to his intractable vomiting.

Spontaneous PR is often asymptomatic. However, neurologic symptoms have been reported [2-6]. Due to the association of spontaneous PR with pneumomediastinum and pneumothorax, patients may report pleuritic substernal chest pain, dyspnea, and neck pain. Patients presenting with signs and symptoms of pneumomediastinum in the setting of maneuvers that increase intrathoracic pressure may warrant neurologic evaluation and CT imaging of the neck and chest to assess for concurrent PR [19]. PR can be anatomically classified into intradural PR or extradural PR [1]. Intradural PR occurs when air is present in the subdural or subarachnoid space, is typically associated with more serious etiologies such as major trauma and is more likely to be symptomatic [12]. With extradural PR, air is present in the epidural space and is usually an incidental finding. Correctly classifying PR as intradural versus extradural may be difficult, though extradural PR tends to accumulate in the posterior extradural space due to the reduced resistance of the connective tissues compared to the anterior vascular network [1].

Currently, there are no management guidelines for spontaneous PR. From our literature review, PR management involves a multidisciplinary team approach and usually resolves with conservative therapy, including bed rest, pain management, and medications to avoid straining, such as anti-emetics, anti-tussives, and laxatives. Close monitoring and serial neurologic exams are encouraged to evaluate the progression of neurologic symptoms. Serial spinal imaging and prophylactic antibiotics were generally not advised unless there was concern for cord compression or infection, respectively [1,12]. In the cases reviewed for this report, follow-up imaging generally showed air reabsorbed in the bloodstream in a matter of days to weeks with little to no recurrence. More aggressive treatments that have been reported in cases of symptomatic spontaneous PR are aimed at addressing the underlying etiology, including laminectomy [4,20] and fistula correction [5].

## Conclusions

Pneumorrhachis is a rare and potentially underdiagnosed finding in clinical practice. Depending on the underlying etiology, most patients are asymptomatic. There are no current guidelines established for managing these patients, leaving a void in patient care. From our literature review and our experience, spontaneous pneumorrhachis management involves a multidisciplinary team approach and usually resolves with conservative therapy. We present our case and encourage others to detail their experiences to aid clinicians in managing these patients until guidelines are established.
